# Editorial for Special Issue “Hydrogelated Matrices: Structural, Functional and Applicative Aspects”

**DOI:** 10.3390/gels11020146

**Published:** 2025-02-19

**Authors:** Enrico Gallo, Carlo Diaferia

**Affiliations:** 1 IRCCS SYNLAB SDN, Via G. Ferraris 144, 80146 Naples, Italy; enrico.gallo@synlab.it; 2 Department of Pharmacy, University of Naples “Federico II”, Via D. Montesano 49, 80131 Naples, Italy

## 1. Introduction and Summary

Gel-based materials have found important applications in fields such as food, healthcare, cosmetics, and bioanalysis [1,2,3,4]. Meanwhile, advances in our understanding of the functional, structural, and manufacturing processes of gels are expanding their potential as tunable elements in fields including energy, soft robotics, water treatment, chemistry, and pharmaceutical sciences [5,6,7,8]. Recent advances have shown that gels can function as dynamic systems with programmable properties, making them highly adaptable to specific requirements across diverse disciplines. For instance, their responsiveness to external stimuli—such as temperature, pH, and light—has expanded their use in the design of smart materials [9,10,11].

Multiscale analyses and the development of supramolecular low-molecular-weight gelators, including peptide-based ones, have broadened the range of soft-gel materials [12,13]. Notably, peptide-based gels have garnered significant attention due to their biocompatibility and potential for sustainable applications, particularly in drug delivery and tissue engineering. The growing interest in gels is also reflected in the expanding variety of gelation-triggering techniques and methods, along with enhancements in rheological properties and shapability. This progress underscores the potential of gels to serve as foundational materials for next-generation technologies, ranging from biomedical devices to environmental remediation systems [14,15,16].

In this context, this Special Issue, entitled “Hydrogelated Matrices: Structural, Functional, and Applicative Aspects” in *Gels*, aims to bring together and report on these complementary aspects of gels, showcasing examples of both polymeric and peptide self-assembling gels. In addition to this Editorial, we are pleased to highlight that the Special Issue features 15 articles in total, reflecting researchers’ interest in the properties, formulations, application scenarios, and characterization of gels. These contributions collectively underscore the growing recognition of gels as versatile and transformative materials across multiple scientific domains, paving the way for Vol II.

The following content provides a brief overview of the diverse topics explored in this Special Issue, highlighting the innovative approaches and breakthroughs in the study and application of gels. These articles exemplify the stimulating interdisciplinary approaches facilitated by gelated matrices.

## 2. Overview

Polyvinyl alcohol (PVA), poloxamer 407 (P407), and vinylcaprolactam (VCL) are among the synthetic polymers evaluated for gel formulation and applicability.

The study by Quispe-Siccha et al. [17] focuses on the development and characterization of PVA capsule-shaped matrices. Two types of PVA, differing in molecular weight, were used as starting materials in a two-step process involving gel preparation and hydrogel cryogelation. Additionally, various PVA concentrations were evaluated. Biocompatibility (tested on the SUP-B15 cell line), porosity, cell proliferation profiles, and diffusion rates were analyzed and correlated with the controlled release of glucose, which was incorporated into the matrices through a post-formulation injection step. By varying both PVA concentration and molecular weight, Ramírez-Chavarría et al. [18] analyzed the physical–mechanical properties and dynamic models of hydrogel matrices with tissue-mimicking profiles. Notably, the photoacoustic response signal was found to influence the hydrogel features, demonstrating a linear relationship between energy absorption capacity, energy transfer, and PVA concentration. The damping ratio and natural frequency were also shown to correlate with both density and elasticity modulus, with progressively increasing values as PVA concentration rose. Overall, the results highlight the potential application of hydrogels as biomimetic platforms for tissue simulation and imaging.

Poloxamer 407 (P407) facilitates gelation in an in situ thermo-gelling approach. To improve the formulation’s adhesiveness and gel strength, P407 was combined with hydroxypropyl methylcellulose to create a vehicle for the nasal drug delivery of chlorhexidine–silver nanoparticle conjugates, as reported by Ivanova et al. [19]. Key functional parameters, such as sprayability, mucosal retention time, washout time, drug release, and antimicrobial activity, were also studied.

Raduly et al. [20] found that Ag^0^–ginger nanocomposites exhibit antimicrobial activity when physically incorporated into hydrogels composed of natural polymers to form gelled matrices. These nanocomposites were integrated into three types of matrices based on rice flour, alginate, and carob flour. The resulting materials were then deposited onto cotton fabrics, producing hydrophilic films with antimicrobial properties, highlighting their potential applications in the textile industry.

Voycheva et al. [21] proposed hydrogels functionalized with mesoporous silica nanoparticles for thermosensitive and pH-dependent drug delivery of doxorubicin. The grafted matrix was synthesized by combining vinylcaprolactam (VCL) and agar using a free radical polymerization approach. Drug delivery efficiency was evaluated under tumor-simulating conditions (40 °C and pH 4.0), achieving complete release within 72 h.

Peptide-based hydrogels constitute an innovative class of biomaterials with tunable properties and excellent biocompatibility, making them highly suitable for applications in biomedicine, tissue engineering, and drug delivery. These hydrogels are formed through the self-assembly of peptides into highly ordered nanostructures, driven by non-covalent interactions such as hydrogen bonding, π-π stacking, and hydrophobic forces. The resulting networks exhibit unique viscoelastic properties and can respond to external stimuli such as pH, temperature, and ionic strength, further enhancing their versatility. The design of peptide hydrogels often involves ultrashort peptides or peptide amphiphiles that capitalize on minimalistic sequences to achieve functional properties.

The study by Jitaru et al. [22] presents the design and characterization of a novel pentapeptide, FEYNF-NH_2_, derived from the hen egg-white lysozyme sequence FESNF-NH_2_. This peptide demonstrated its ability to self-assemble into β-sheet-driven dendritic structures under physiological conditions, while fluorescence analysis identified a quenching phenomenon at two peptide concentrations. Microscopic techniques, including POM and TEM, revealed the complex framework of the self-assembled structures, indicating their potential to form gel-like materials. In silico studies confirmed the β-sheet arrangements and further revealed non-covalent interactions between the FEYNF peptide’s protonated dimer and the Drew–Dickerson dodecamer DNA sequence, a widely used model in structural biology. This finding underscores the peptide’s potential for DNA recognition, linking peptide self-assembly with nucleic acid interactions.

Gallo et al. [23] broadened the scope of Fmoc-FF-based hydrogels by incorporating a lysine (K) residue at the C-terminus of the peptide sequence, introducing a functional amino group with potential for derivatization with bioactive molecules. The study investigated the properties of the Fmoc-FFK tripeptide both individually and in combination with Fmoc-FF at various weight ratios (1:1, 1:5, 1:10, 1:20 *w*/*w*). The Fmoc-FFK monomer exhibited self-assembly and gelation above a concentration of 1.0 wt%, forming a soft hydrogel (G′ = 24 Pa). The incorporation of Fmoc-FFK into Fmoc-FF significantly improved the mechanical properties of the multicomponent hydrogels while preserving the structural organization and morphology of the pure Fmoc-FF hydrogel. Biocompatibility tests, conducted in vitro on HaCaT and 3T3-L1 cell lines, yielded favourable results for all proposed systems.

Buzzaccaro et al. [24] demonstrated the production of homogeneous and transparent gels using (LDLK)_3_-based SAPs at concentrations ranging from 1 to 10 g/L, controlled via a urea–urease hydrolysis reaction. The method allows independent regulation of the final pH (via urea concentration) and gelation rate (via urease concentration). At high SAP concentrations, rigid gels are formed, which immobilize nanoparticles, while lower concentrations produce flexible gels with partial nanoparticle mobility. These adaptable morphologies are promising for multi-drug release, tissue regeneration, and 3D cell culture applications. The slow gelation process enhances scaffold reproducibility, enables the inclusion of chemotactic agents, and provides insights into gelation mechanisms through light scattering. The approach can be extended to other gelators, offering promising applications in biomaterials and regenerative medicine.

Natural polymers offer numerous advantages for hydrogel formation, making them valuable across biomedical, environmental, and industrial fields. Key benefits include biocompatibility and biodegradability, ensuring safe interaction with biological systems and eco-friendly degradation. Moreover, as they are derived from renewable resources, these polymers are sustainable and cost-effective.

The manuscript of Machado et al. [25] demonstrates that encapsulation in calcium–alginate-based hydrogel is an effective strategy for delivering Akkermansia muciniphila. The technique produced high-loaded capsules that maintained probiotic viability at ~10^8^ CFU/g during 28 days of refrigerated aerobic storage. The encapsulating hydrogels exhibited greater viability and stability under in vitro gastrointestinal conditions compared to free cells, suggesting that the method mitigates the adverse effects of prolonged storage and subsequent gastrointestinal transit. Furthermore, the encapsulation ensured delivery levels exceeding the probiotic threshold (10^6^ CFU/g) even after storage and gastrointestinal passage.

Loi et al. [26] developed linear polycaprolactone substrates with microchannels, where a fibrinogen-based hydrogel containing C2C12 cells was deposited to examine the impact of the co-printing technique on cellular behavior. They assessed cell viability and differentiation over a 21-day culture period, reporting significant enhancement of differentiation at 14 days. The study also evaluated the viability and differentiation of C2C12 myoblasts on distinct geometries, demonstrating that the linear pattern outperformed the others in promoting C2C12 myotube differentiation. After 14 days in culture, C2C12 cells in the linear structure fused to form aligned myotubes, particularly at the edges of the structure, with elevated expression levels of skeletal muscle markers such as MyoD.

Enache et al. [27] used sodium carboxymethyl cellulose (CMC) to create ionically cross-linked hydrogels in the form of magnetic beads by incorporating MnFe_2_O_4_ nanoparticles and SDS surfactant to enhance adsorption capacity and stability. The beads were characterized and evaluated for their adsorption properties. The adsorption followed the PFO model and Langmuir isotherm, indicating spontaneous, exothermic adsorption. The beads demonstrated 93% desorption efficiency and could be reused. Molecular docking revealed that the interaction between the beads and MB dye was electrostatic. These CMC-based hydrogels are promising sorbents for environmental applications due to their biocompatibility, high adsorption capacity, and recyclability.

The study conducted by Materni et al. [28] examined the preventive effect of the sunflower oil-based ozone gel Ozosan^®^ on dry sockets through a double-blind, split-mouth, randomized placebo-controlled clinical trial involving 200 patients. The results indicated that Ozosan^®^ effectively reduces the incidence of dry sockets, following the extraction of an inferior third molar. Furthermore, no adverse effects or noticeable differences in the post-extraction wound healing process were reported. This promising result in preventing alveolitis encourages further randomized studies on treating periodontitis and other inflammatory oral diseases.

A cross-linked gel polymer electrolyte (C-GPE) based on epoxidized soybean oil (ESO) was synthesized by Zhang et al. [29] using an in situ thermal polymerization with lithium bis(fluorosulfonyl)imide (LiFSI) as an initiator. The macroscopical organization of the cross-linked structure was found able to increase the ionic conductivity and ion mobility. The wide electrochemical window (of up to 5.19 V vs. Li^+^/Li), along with high ionic conductivity, super-low glass transition temperature, and good interfacial electrodes/electrolyte stability, demonstrated the potential of these matrices in improving anode stability by adjusting the distribution of electrolyte on electrode surfaces in lithium gel polymer batteries.

The effect of pressurization and extraction from natural acorns was evaluated by Castro et al. [30] Significant structural and property changes, in terms of span distribution and uniformity, were detected in relation to amylose/amylopectin ratios. However, no substantial differences were found in parameters including gelatinization temperatures, relative crystallinity, and polymorphism. Acorn starch matrices also exhibited lower gelatinization temperatures, enthalpies, in vitro digestibility, superior pseudoplastic behavior, and lower resistance to deformation with respect to commercial starch, thus suggesting the potential for the acorn starches as a novel food ingredient.

The study of Kim et al. [31] focused on the development of a gel-based drug delivery system aimed at improving colon cancer treatment by incorporating 6-mercaptopurine (6-MP), an anticancer drug, into a thiolated gelatin/polyethylene glycol diacrylate matrix (6MP-GPGel). The system demonstrated a controlled, sustained release of 6-MP, with enhanced release rates in acidic or glutathione-rich environments simulating a tumor pH. In vitro results showed that while pure 6-MP allowed for cancer cell proliferation after five days, the 6MP-GPGel formulation effectively suppressed cell survival continuously. The study concludes that the 6MP-GPGel system offers a promising, minimally invasive, and localized drug delivery approach for colon cancer therapy, potentially improving treatment outcomes.
